# ‘Since people who have mental illness are stigmatised, their service is also stigmatised. You get a massive hospital building and there is no mental health facility’: exploring perceptions of mental health, stigma of mental illness, care-seeking and service use in the Somali Regional State of Ethiopia

**DOI:** 10.1192/bjo.2026.11016

**Published:** 2026-04-13

**Authors:** Nasir Warfa, Charlotte Hanlon, Elyas Abdulahi, Mohamed Abdi Wali, Abdifetah Abdullahi Sheikh, Medhin Selamu, Mussie Abdosh Hassen, Fowsia Abdulkadir Hashi, Abdulahi Hussein, Bashir Abdulahi, Ahmed M. Abdinasir, Abdikarim M. Abdi, Mustafa al’Absi, Roxanne Keynejad, Solomon Teferra, Chris Willott

**Affiliations:** Institute of Health Science, Jigjiga University, Ethiopia; Centre for Clinical Brain Sciences, Division of Psychiatry, University of Edinburgh, UK; Research and Community Services, Jigjiga University, Ethiopia; Department of Research, Publication and Ethics, Research Ethics Committee, University of Kabridahar, Ethiopia; Epidemiology and Biostatistics, College of Health Science, University of Kabridahar, Ethiopia; World Health Organization, Addis Ababa, Ethiopia; Disease Prevention and Disease Control, Somali Region State Health Bureau, Jigjiga, Ethiopia; Commission for Human Rights, Jigjiga, Ethiopia; Health Science, Jigjiga University, Ethiopia; College of Business, Department of Management, Jigjiga University, Ethiopia; Administration and Business Department, Kebridahar University, Ethiopia; Faculty of Pharmacy, Yeditepe University, Türkiye; Department of Psychiatry, Addis Ababa University, Ethiopia; Behavioral Medicine Laboratories, University of Minnesota Medical School – Duluth Campus, Minnesota, USA; Health Service and Population Research Department, Institute of Psychiatry, Psychology & Neuroscience, King’s College London, UK; Department of Population Health Sciences, Faculty of Life Sciences and Medicine, King’s College London, UK

**Keywords:** Stigma of mental illness, religious and traditional treatment, transcultural psychiatry, mental health services, Somali cultural diagnosis of mental illness

## Abstract

**Background:**

There are few mental health services in the Somali Regional State (SRS) of Ethiopia, and many people with mental health conditions turn to traditional healing. Also, little is known about perspectives on mental ill health and care in this sociocultural context.

**Aims:**

The study explores the experiences and manifestations of mental health-related stigma in the SRS, to inform the development of mental healthcare systems.

**Method:**

We conducted 16 semi-structured interviews with health workers, aspirational leaders, users of mental health services and carers in Jigjiga and Kabridahar, two cities in the SRS, between April and July 2024. Translated transcripts were imported into NVIVO version 14 for coding and were then analysed using the thematic analysis method. We identified three main themes: (a) mental health stigma, (b) societal neglect and (c) misunderstanding of mental ill health.

**Results:**

Participants suggested that most people in the SRS view mental health in binary terms, in which a person is either ‘mad’ or sane; a corollary is that only severe conditions with overt behavioural manifestations were viewed as mental illness. Most people viewed mental health conditions as having spiritual causes. Mental health stigma was reportedly widespread and severe. These barriers contribute to care-seeking that is delayed and initially focused on faith-based providers.

**Conclusions:**

Any intervention to improve the provision of mental health services and the development of mental health systems must take into account the perspectives of service users and carers, and address the widespread stigma and lack of knowledge around mental illness.

There are three key questions in this study:

- What is already known on this topic?
  Ethiopia’s Somali Regional State (SRS) is emerging from four decades of war and conflict. The SRS has specific characteristics and challenges that include the widespread role of Islam in the conceptualisation and treatment of mental ill health, culturally rooted stigma towards mental health conditions and the impact of a severely underdeveloped mental health system.
- What does this study add?
  The endurance of harsh life situations, traumatic experiences and mental health conditions has not been matched by meaningful clinical interventions, and little attention has been paid to the needs of people with mental health conditions, partly because of pervasive stigma in the SRS.
- How might this study might affect research, practice or policy?
  Stigma is intrinsic to the experiences of mental illness in the SRS. It structures every aspect of service users’ lives and affects personal relationships, employment opportunities for meaningful participation in society and the ways in which the person discloses and seeks care. Recognising the unique clinical needs of service users and carers in resource-limited settings (such as the SRS) is essential because researchers, practitioners and policy-makers cannot intervene to foster positive change in any clinical service development without first understanding the core obstacles to change, and what people with mental health conditions want to change.

Ethiopia is the second-most populous country in Africa, with an estimated population of 132 million.^1^ The SRS is the second-largest state in Ethiopia in terms of size and has a population of over 6 million people.^2^ Since 2018, following 40 years of recurrent conflict, the region has been stable and has experienced a period of rebuilding and renewal.^3^ Nonetheless, the influence of previous conflicts, poverty, substance misuse and lack of mental health infrastructure has impacted population mental health.^4^

There are very few mental health professionals in the SRS, with limited out-patient services provided at a only few general hospitals. In 2022, one psychiatrist and approximately 20 psychiatric nurses covered the region.^5^ Efforts to integrate mental health into the 250 primary health centres in the SRS are under way. However, multiple factors have constrained the development of a people-centred healthcare system in the SRS, of which stigma is key. Culturally and historically rooted stigma around mental health conditions across the SRS hamper the quality of, and access to, mental health services. Culturally rooted stigma towards mental health conditions was an obstacle to implementing the World Health Organization (WHO) mental health Gap Action Programme intervention guide in the SRS.^3^ Lack of mental health help-seeking at primary care facilities impeded its provision, leading people with mental health conditions to rely on traditional practices and faith-based organisations.^3^

The SRS has specific contextual characteristics that affect mental health and mental health service provision. These include widespread consumption of *khat* (a stimulant leaf whose active ingredient, cathinone, has amphetamine-like properties); the role of Islam in the conceptualisation of mental ill health; years of war and conflict; climate change-related hardship, migration; displacement; and a severely underdeveloped mental health system.^3,6^

## Aims

The primary aim of this study was to explore perceptions of mental health conditions and experiences and manifestations of mental health-related stigma in the SRS, in order to inform the development of the mental healthcare system. We focused on stigma because previous evidence from Ethiopia suggests that it is a pervasive concern.^3–9^

## Method

We conducted 16 in-depth interviews in the SRS capital, Jigjiga, and in the third-largest city, Kabridahar, between April and July 2024. Building on our prior research on the views of SRS policy-makers,^3^ we sought to analyse the perspectives of service users, carers and front-line staff who work with people with mental health conditions, with the support of stakeholders from the Somali Regional Health Bureau (SRHB).

We used a purposive sampling strategy to identify people with experience of providing or using mental health services in the SRS. Eight participants were from Jigjiga and eight from Kabridahar. Interviews were conducted with primary healthcare staff, mental health professionals, a youth worker, a pharmacist, aspirational leaders and people with mental health conditions and their carers (Table 1).

**Table 1:** Demographic data of participants

Gender	Role in research	Location	ID number
Male	Pharmacist	Health bureau	01
Female	Psychiatrist	Public hospital	02
Male	General practitioner	Public hospital	03
Female	Mental health volunteer	Public university	04
Male	Psychiatric nurse	*Ilaaj*	05
Female	Nurse	Public hospital	06
Male	Service user	Charity clinic	07
Male	Service user	Charity clinic	08
Male	Service user	Charity clinic	09
Male	Service user	Charity clinic	10
Male	Carer	Charity clinic	11
Male	Nurse	Charity clinic	12
Male	Carer	Charity clinic	13
Male	Psychiatric nurse	Charity clinic	14
Male	Nurse	Charity clinic	15
Male	Non-specialist doctor	Public hospital	16

All participants were over 18 years of age and were able to provide informed consent to participate in the study. Participants were screened for eligibility and recruited through primary healthcare facilities, charity clinics, universities and *ilaajs*. *Ilaajs* (or *cilaajs*) are in-patient mental health facilities that provide religious (Islamic) and biomedical services. In addition, charity-run clinics refer people to in-patient mental health facilities also run by charities or non-governmental organisations, often with a religious dimension. They provide spiritual and limited biomedical care to service users.

The study combined both inductive and deductive approaches to data collection and analysis. The topic guide included questions about stigma and mental ill health. Perceptions of neglect and misunderstanding of mental ill health (particularly by service providers) was something that emerged from the data rather than being a result of the questions asked.

### Ethical standards

Participants were screened for eligibility by trained, supervised data collectors before being invited to provide written and informed consent. In the event that a participant was unable to provide written consent, they provided a thumbprint rather than a signature. We prioritised participants’ well-being at all times. The information sheet (also read verbally) explained that participants could pause or stop the interview at any point. Any participants experiencing distress would be offered support by local mental health services.

### Data analysis

Interviews were recorded in both the Somali and English languages, translated and transcribed in English by the two native Somali-speaking researchers (N.W. and A.M. Abdinasir). Collected data were imported into NVIVO version 14 for macOS (Lumivero, Denver, Colorado, USA; https://lumivero.com/products/nvivo/) for description-focused coding. We analysed the findings using the thematic analysis method.^10^ There are several approaches to the thematic analysis process, including familiarisation with the data, searching and coding key texts and identifying interested themes and then indexing them.^10^ For example, we searched, selected and coded the relevant statements and sentences, which we then used to produce three broad themes: (a) mental health stigma, (b) societal neglect and (c) misunderstanding of mental ill health. Finally, we selected the relevant quotes to illustrate and unpack the meanings behind these indexed core themes.

### Findings

This manuscript focuses on the three aforementioned related categories: (a) mental health stigma, (b) societal neglect and (c) misunderstanding of mental ill health.

### Societal neglect and misunderstanding of mental illness

This issue was particularly highlighted by the professional participants, who frequently described the ways in which society treats people with mental health conditions (without being prompted). By contrast, service users did not mention this and tended to focus more on their own experiences. Service providers were much more likely than service users to be critical of others regarding their views on mental health.

Service providers provided two distinct perspectives on the conceptualisation of mental ill health: their own views and their views on how the issue was perceived among their patients and others in SRS society. For most health workers in our research, mental health was conceptualised using a biomedical model. One respondent quoted the WHO definition of mental health, while another suggested that it was ‘just like other health problems’. For another participant, however, the perception was quite different: mental ill health was something severe, often with obvious behavioural manifestations that violated known societal norms and values, such as a naked person roaming around the streets:

‘When we talk about mental illness, what comes to my mind is a naked person on the street and uncared for or someone who is chained because they may kill people. Number one, that is what comes to mind.’ (Male, pharmacist, participant 01)

Although most professionals conceptualised mental health based on their education and training, they perceived the views of community members differently. Most service providers felt that understanding of mental health was low and characterised by the belief that mental health is binary: one is either mad/crazy (*waali*) or not.^11–17^ This is a common Somali view of mental ill health, and means that mild and moderate conditions are infrequently conceptualised as ‘mental illness’. Only when the person’s condition deteriorates substantially, causing them to become homeless or pose risks to others (or the family perceive that they need to be chained up), is mental ill health acknowledged:

‘There are many different categories of mental health problems, like depression, anxiety, PTSD [post-traumatic stress disorder], OCD [obsessive–compulsive disorder], psychosis, schizophrenia and so on. But in our culture, they only believe mental illness when they become schizophrenia, that is when they acknowledge mental illness, it is when the person goes mad.’ (Male, non-specialist doctor, participant 16)

When respondents were asked about their perceptions of mental ill health, they invariably focused on serious manifestations such as psychosis and schizophrenia, and these were associated with death:

‘The society believes this illness is just like the step before death, or like death. The person cannot face the society because they are stigmatised and discriminated. The person believes that, in this world, today, you are nothing. That is the stage the person with mental illness is at … Our society do not know where mental illness starts, just where it ends.’ (Female, mental health volunteer, participant 04)

Alongside the perception of mental health as being embodied only by something very serious was the view that it was also incurable:

‘I remember my grandmother saying that a man who becomes mentally ill may feel OK for a bit but will always be ill. This is what our grandparents believed about mental illness. I mean a mad man or a man with brain/head problems may calm down but never be cured*.’* (Male, psychiatric nurse, participant 05)

‘The society, however, thinks people can’t be treated and that makes people to hide their conditions… Some people do not believe there is treatment for mental illness and that there are no normal treatment places*.’* (Female, mental health volunteer, participant 04)

Another respondent described physical health as *‘*normal health’ in contrast to mental health, illustrating the degree to which mental health is considered an abnormality rather than a ‘normal’ health condition.

A number of participants reported the idea that people with mental health conditions ‘lack insight’ and do not understand that they are suffering from a mental health condition:

‘Most of my patients have severe mental illness and they are in denial. And usually, they don’t have insight. They don’t understand what is happening to them … Most of them they don’t know or understand their illness*.’* (Female, nurse, participant 02)

This lack of insight was linked to the dearth of information about mental health within society:

‘It is lack of awareness, and no one gives them awareness, and it could be the myths they believe, but we only realise someone is mentally ill when they become schizophrenic.’ (Male, non-specialist doctor, participant 16)

Lastly, many participants narrated a spiritual dimension of mental ill health, particularly the view that it was caused by ‘*jinn’* or evil spirits. However, what is interesting to note is that healthcare providers were less likely to state that they believed this themselves, rather that this was how they perceived that ‘society’ conceptualised the causes of ‘mental illness’.

Participants suggested that mental health conditions are caused by substance abuse, notably chewing *khat*, drinking alcohol and smoking cigarettes, and by life experiences such as abuse or neglect. One service user discussed spiritual causes such as ‘evil eye’ and ‘a curse from your parents’. Multiple service providers ascribed this view to others:

‘They don’t believe that there is such thing as mental illness and there is mental health service. They just believe things like jinn … So, they will not bring people with mental health conditions to whatever service that is here.’ (Male, non-specialist doctor, participant 16)

‘People believe jinn causes mental illness 100%. They believe that from all the problems that cause mental illness, jinn is number one. That is why they take the person to Sheikh [religious leader].’ (Male, psychiatric nurse, participant 05)

As alluded to in the above quote, this conceptualisation of mental ill health has an impact on care-seeking behaviours. These decisions are not usually made by the person themselves, but by their family or the person responsible for them. Because most people perceive that mental illness has a predominantly spiritual cause, is binary and refers only to something serious, this has two specific effects on care-seeking.

The focus on spiritual causes of mental distress means that care-seeking follows this conceptualisation: if a problem has a spiritual cause, only a spiritual solution can address it. This means that the first choice of provider is invariably religious and/or spiritual rather than biomedical, and this therefore delays critical care (and sometimes leads people to becoming exposed to harmful practices):

‘Some of the people believe that if someone suffers from witchcraft only religious people can treat them, using things like plants and the readings of the Quran. And there are places where people are used with host water or get beaten and then a voice will come out from within the person, telling where they got witch-crafted and got ill. I also hear a place where they use electricity against people and/or expose them to smoke so then the things that possessed them leaves the body.’ (Male, nurse, participant 12)

‘Some people they manage [jinn] well using religious treatment, and they read Quran on the person until the jinn may leave the body. However, others they misuse this system and sometimes torture the person, so they try to force jinn to leave the body by beating them. There are also people who use traditional medicine and cultural plants to treat jinn.’ (Male, pharmacist, participant 01)

A common situation is for someone with mental health conditions to try many traditional methods of treatment before eventually seeking to access mental health services:

‘As soon as the person gets [possessed by] jinn, they take them to the religious people for treatment. I have seen many patients in this situation. After they got tired with other traditional treatments, they bring them here and say we have taken them to many Sheikhs … They said when the religious treatment hasn’t worked, we thought let us give the hospital treatment a chance.’ (Female, nurse, participant 06)

‘From the patient’s side, they associate their illness with some spiritual forces. Or spiritual entities. They think or they believe their illness has to do with some evil spirits. Because of this, they seek help from spiritual leaders. They consider medical treatment as a last resort.’ (Female, psychiatrist, participant 02)

The conceptualisation of mental illness as binary, and the inclusion of only serious illness means that care-seeking, particularly for biomedical solutions, often happens very late:

‘They try their best to avoid seeking treatment … They don’t believe that there is such thing as mental illness and there is mental health service … I heard a situation where a mother had a son who got ill and was chained under a tree for 15 years in a rural place … [An activist] washed him, fed him, clothed him and received treatment … Then, they brought back his sanity and then took him to his mother, and she couldn’t believe how well he was. He was suffering for fifteen years for something that could have been treated.’ (Male, non-specialist doctor, participant 16)

Mostly, the professional participants suggested that local conceptualisations of mental health conditions had detrimental effects, particularly on care-seeking. Reluctance to seek biomedical care for mental health conditions was compounded by stigma.

### Mental health stigma

Mental health stigma was acknowledged by all participants in the SRS as a pervasive issue, with implications for those suffering from mental health conditions and their families. Most people discussed stigma in relation to mental health conditions with visible physical and behavioural manifestations:

‘Stigma, it is the biggest contributor to mental illness. In our society, what takes a minor symptom to a severe condition is our society. If they hear anything about mental illness, they say it is crazy, he is crazy. So, a minor condition turns into a major disorder because of our society’s stigmatisation of mental illness.’ (Male, non-specialist doctor, participant 16)

Participants described how mental health stigma manifests in various ways, including violence and abuse, which frequently lead the person and their family to isolate themselves from society. Stigmatised terms include ‘muddy person’ (*kii dhoobada ahaa*), ‘mad person’ (*kii waalnaa*) and ‘the one who is lost’ (*kii lumay*). One participant commented:

‘Some people attack them violently with stones, mostly children do this. People find it difficult to understand mental illness and they have different beliefs about it since they are not health professionals. And there are others who stigmatise and laugh at them.’ (Male, psychiatric nurse, participant 14)

Participants expressed the view that isolation of people with mental health conditions takes a variety of forms. The most widely discussed was hiding away at home: one respondent told of a patient who concealed her illness from everyone, including her adult children:

‘Lots of people they are hiding their illness. I know this woman she was ill, but she didn’t want her children to know her illness. She said she can manage this by herself, and the children will worry more about her … they will panic … and they themselves will get mental health problems, and may talk to themselves, she was afraid of that. She said I know my illness, and I can take my medication, and they don’t need to know about this.’ (Female, nurse, participant 06)

According to participants, it is common for people with mental health conditions to be kept outside society:

‘It affects [people with mental health conditions] a lot because they are stigmatised and isolated and are no longer with the community. They don’t get the services they need, and so they end up with nothing and they have nothing because of stigma and not being able to be trusted with anything. It is believed that they cannot do anything.’ (Male, psychiatric nurse, participant 05)

Because of the stigma of mental ill health, people with mental health conditions would not be considered for positions of authority, and were excluded from ordinary facets of life including marriage and employment:

‘If a person is slightly suspected of having mental illness … they are given no responsibility. They are given no work, there is no trust … Even if there is a family business, they will not be trusted to work for them. They are refused everything. Refused work, refused to be given any role and they are just told… you are mad, so that person would go mad, and leaves the society.’ (Male, pharmacist, participant 01)

‘When someone becomes mentally unwell, they get stigmatised, and the person will not find employment as no one going to trust them. They will not be able to get married. They say why marry a woman to a mad man. They say there is too much going on with this person and cannot take a full responsibility.’ (Male, psychiatric nurse, participant 05)

These comments suggest a situation in which people with mental health conditions are treated as if they have no capacity to make decisions for themselves, are not seen as full members of society and are kept isolated from others. In this context, we can explicitly say that stigma *per se* provokes mental ill health.

Mental health stigma has another impact that contributes to the perpetuation of the issue: fear of the consequences of seeking care – such as being seen by others or treated badly by health workers – leads people to avoid or hide care-seeking altogether:

‘They don’t want to be recognised or labelled as an individual with mental illness, they are less likely to come to OPD [out-patient department]. Even if they must, they cover their face with mask or veil so then their face is unrecognised, so then they are not labelled as mentally ill. It gets in the way of everything.’ (Female, psychiatrist, participant 02)

‘It doesn’t matter if a person has mental health problems or not, people like to go to a place where they feel they are welcomed. However, if you believe there is a place where you are going to be welcomed with insults or people are not going to serve you, then you won’t go to that place.’ (Male, psychiatric nurse, participant 05)

From participants’ perspectives, mental health stigma causes under-investment in the mental health system because mental health services are treated in the same way as people with mental health conditions. In a nutshell, mental health services are themselves stigmatised:

‘Since people who have mental illness are stigmatised, their service is also stigmatised. You get a massive hospital building and there is no mental health facility. So, the stigma is not just about people, but about services too … They think these people are hopeless so, why should I spend money on their service? And it is better if I spend the money on other illnesses.’ (Male, pharmacist, participant 01)

## Discussion

Participants identified relationships among community conceptualisations of mental ill health, its origins and character, stigmatisation within society and the care-seeking process. Mental health was predominantly viewed in two ways: (a) as a binary between madness and sanity, in which ‘mental illness’ was framed only as a very severe condition (madness), and non-psychotic illnesses such as depression were not viewed as mental illness at all; and (b) as something with predominantly spiritual causes, including witchcraft, *jinn* and curses.

Mental health stigma is pervasive and has detrimental effects on people with mental health conditions and their families. Many people isolate themselves from society rather than facing the consequences of stigma, leading to late and inappropriate care-seeking, exacerbation of mental health symptoms and underinvestment in the health system. All of these impacts contribute to a cycle of mental illness, stigma and poor-quality services.

Given the importance of stigma in our study, it is interesting to map the binary conceptualisation (madness/non-madness) of mental illness onto definitions of two types of stigmatised person: the discredited and the discreditable.^18^ Goffman^18^ characterised stigma as ‘[An] attribute that is deeply discrediting and that reduces the bearer from a whole and usual person to a tainted and discounted one’. This characterisation would certainly reflect the experiences of having mental health conditions in the Somali culture.

For Goffman, the discredited is someone for whom the stigmatising attribute exists and can be seen by others; the discreditable, by contrast, suffers from the stigmatising attribute but can hide it from others. The characterisation of a discredited person would correspond to the experience of people in the SRS who suffer from psychosis and schizophrenia and are visibly distressed. An example would be the person with matted hair and no clothes (or someone wearing muddy, dirty clothes), and at whom children throw stones. The definition of the discreditable person could refer to someone suffering from depression or anxiety and who is able to conceal their condition. In the context of high stigma, there are clear motivations for someone who is discreditable (rather than discredited) to camouflage their condition, given its implications for multiple aspects of their lives. In turn, this makes mental healthcare-seeking a risky proposition, because being seen at an out-patient mental health clinic raises the risk of being ‘outed’ as someone with a mental health condition.

From the findings of this study, there were strong perceptions that mental health conditions are incurable, which, combined with high levels of stigma, create difficulties for people discredited ever to regain their non-stigmatised status. Most people with severe mental health conditions who recover from their condition do so through long-term engagement with care, which is itself highly stigmatised in Ethiopia.^19^

The marginalisation of service users is a key element of mental health stigma, and a reason to seek greater involvement of service users in the development of interventions to improve services. Pervasive stigma is a barrier to the realisation of this goal, because people suffering from mental health conditions are generally perceived to be of low status and lacking the opportunity to – or being directly prevented from – making meaningful contributions to society.^20^ By contrast, however, in the same study, participants perceived various potential benefits to the inclusion of mental health service users in health system strengthening, including reducing marginalisation and improving services through increased voice.^20^ In the SRS, we are actively seeking to emulate this approach in involving mental health service users in strengthening the system.

The marginalisation of mental health service users affected recruitment into this study; also, their reluctance to fully express their views and preferences has been documented elsewhere in Ethiopia.^21–23^ This has been remedied through interventions to empower service users and reduce stigma,^21–24^ and via the growing prominence of service user associations (for example, Ethiopia’s influential Mental Health Service User Association). We hope to replicate these or similar interventions in future work in the SRS.

The binary conceptualisation of mental health – viewing only psychotic illness as ‘mental illness’ – echoes various literature focused on other parts of Ethiopia. Menberu et al^25^ comment that, in their study, over half of the participants did not seek professional help because they did not believe depression was an illness that could be treated. Likewise, Bhui et al^11^ reported that no Somali community members identified depression as a mental illness, arguing instead that it was ‘part of a normal reaction to the stresses of life’. Lastly, Alem et al^26^ suggested that ‘one has to display behaviour that attracts public attention to be recognized as mentally ill’.

These views exacerbate late care-seeking because sufferers (and carers) do not perceive that they are suffering from an illness that is a treatable condition. We found that spiritual conceptualisations of mental health conditions mean that people first seek care from traditional or religious providers and visit biomedical care services only once other options have been exhausted. Wider reasons for lack of engagement with mental health services in primary care include poverty, unreliable medication supplies, the long-term nature of the illness and difficulty in accessing health facilities.^19^ Fekadu et al^27^ identified an equity gap in access to mental health services, with more highly educated people being better able to access these services.

We previously provided evidence^3^ that there has been a reduced focus on mental health service development in the SRS in recent decades. This is partly because of the high stigma attached to mental illness, as one of the study participants put it so eloquently: ‘Since people who have mental illness are stigmatised, their service is also stigmatised’. Not only is their service stigmatised, but funding for the development of a basic mental health system for the population is also stigmatised. For example, the vast majority of people with mental health problems access services through faith-based and traditional health systems, and women with mental health conditions hardly ever use these alternative service provisions.^3,5^

Taken together, the findings from our study support the existence of relationships among cultural understandings of mental ill health, mental health stigma, help-seeking from traditional and spiritual providers and delayed care-seeking (and care provision). Finally, the findings demonstrate the importance of engaging with the Somali cultural models of distress and help-seeking behaviour, which can strengthen the adaptation of psychiatric and clinical psychology interviews and diagnostic measures,^11,12,28^ as well as improving future mental health studies conducted with similar target populations.

### Limitations

Given the limited availability of mental healthcare, and the deeply rooted stigma attached to mental illness, in the SRS we found it difficult to recruit service users and their carers. Also, due to the power differentials that inhibited the elaboration of service users’ responses, our findings are dominated by the perspectives of the professional participants. Barriers to service user expression and their participation in research point to the pervasive effects of stigma. There is therefore scope to broaden the evidence base through co-production of future qualitative studies that include people with lived experience.

We identified a binary conceptualisation of mental health in the SRS in which only severe illness with overt behavioural manifestations is recognised. We found a predominant understanding of mental ill health as having spiritual origins. Pervasive mental health stigma across society means that people frequently conceal mental health conditions for fear of ostracism, rejection, violence and abuse.

The SRHB and King’s College London are working to improve the accessibility and quality of mental health services in the SRS. Engagement with local communities, including people with mental health conditions and their carers, will be essential to ensure that system changes enhance the likelihood of services being accessed. Such involvement and collaboration with the community will be critical in overcoming stigma and engaging with cultural conceptions, stigma and their impacts on care-seeking.

## Supporting information

10.1192/bjo.2026.11016.sm001 Warfa et al. supplementary material Warfa et al. supplementary material

## Data Availability

All data relevant to this study are included in the manuscript. Unpublished interview data can be accessed by contacting the corresponding author.

## Supplementary material

The supplementary material is available online at https://doi.org/10.1192/bjo.2026.11016
