# Supplementary material for: ‘Since people who have mental illness are stigmatised, their service is also stigmatised. You get a massive hospital building and there is no mental health facility’: exploring perceptions of mental health, stigma of mental illness, care-seeking and service use in the Somali Regional State of Ethiopia
Source: BJPsych Open. 2026 Apr 13;12(3):e106. doi: 10.1192/bjo.2026.11016 (PMC13107296; doi:10.1192/bjo.2026.11016)
Supplement: Warfa et al. supplementary material [file S2056472426110163sup001.docx]

**Topic Guide**

**Semi-Structured Questionnaire on perceptions of mental health and mental health service utilisation (Section relevant to this manuscript)**

- Interviewed Person:
- Interviewee
- Time/Place of the Interview:
- Gender/Age:
- Place of birth/Marriage Status:
- Educational status
- Employment status

**Transition Questions**

We would like to talk to you about your general perception of mental illness. Please note that these questions are not about your mental health but about your perceptions of mental illness.

**Questions on perceptions of mental health**

- When you think about mental health, what comes to your mind?
- What kind of issues do you associate with mental health?
- What is your understanding of Jin?
- Do you think Jin causes mental illness?
- What else can you think of may be the cause of mental illness?

**Questions on mental health service utilization**

Now we would like to talk to you about where people who have mental health problems seek treatment. Please do not tell us about their names or identity.

- Do you know people who have mental health issues?
- What kind of mental health issues do they have?
- How long did they have mental health?
- What do they say are the cause of mental health issues?
- Have they sought treatment for mental health services before?
- If yes, what type of treatment have they sought, and from where?
- If no, why not?

**Questions on stigma of mental health**

- What do you understand about stigma?
- How stigma affected on your mental health?
- How stigma affected your life?
- Do you think stigma plays role in treatment avoidance?
- How?
- Do you think stigma stops you from seeking treatment altogether?

**Questions on family and social support**

Here, we would like to know how the family and friends support people who have mental health problems, generally.

- Do people who have mental health problems get support from family or friends?
- What kind of support do they get?
- How long do they get support from family or friends?
- Is this support considered helpful by people with mental health problems?

**Final question:** Is there anything else you would like to share with us about mental health and service use?
